# Night‐to‐night variability of sleep apnea detected by cyclic variation of heart rate during long‐term continuous ECG monitoring

**DOI:** 10.1111/anec.12901

**Published:** 2021-10-18

**Authors:** Junichiro Hayano, Emi Yuda

**Affiliations:** ^1^ Heart Beat Science Lab, Co., Ltd. Sendai Japan; ^2^ Nagoya City University Nagoya Japan; ^3^ Center for Data‐driven Science and Artificial Intelligence Tohoku University Sendai Japan

**Keywords:** day‐to‐day variation, heart rate dynamics, heart rate variability, Holter ECG, intraweek variation, long‐term ECG monitoring, predictive accuracy, sensitivity, specificity

## Abstract

**Background:**

Sleep apnea is common in patients with cardiovascular disease and is a factor that worsens prognosis. Holter 24‐h ECG screening for sleep apnea is beneficial in the care of these patients, but due to high night‐to‐night variability of sleep apnea, it can lead to misdiagnosis and misclassification of disease severity.

**Methods:**

To investigate the long‐term dynamic behavior of sleep apnea, seven‐day ECGs recorded with a patch ECG recorder in 120 patients were analyzed for the cyclic variation of heart rate (CVHR) during sleep periods as determined by a built‐in three‐axis accelerometer.

**Results:**

The frequency of CVHR (Fcv) showed considerable night‐to‐night variability (coefficient of variance, 66 ± 35%), which was consistent with the night‐to‐night variability in apnea‐hypopnea index and oxygen desaturation index reported in earlier studies. In patients with presumed moderate‐to‐severe sleep apnea (Fcv > 15 cph at least one night), it was missed on 62% of nights, and on at least one night in 88% of patients. The CV of Fcv was negatively correlated with the average of Fcv, suggesting that patients with mild sleep apnea show greater night‐to‐night variability and would benefit from long‐term assessment. The average Fcv was higher in the supine position, but the night‐to‐night variability was not explained by the night‐to‐night variability of time spent in the supine position.

**Conclusions:**

CVHR analysis of long‐term ambulatory ECG recordings is useful for improving the reliability of screening for sleep apnea without placing an extra burden on patients with cardiovascular disease and their care.

## 1 INTRODUCTION

Sleep apnea is common among patients with cardiovascular diseases and a factor that could cause the diseases and worsen the prognosis. Sleep apnea causes repeated nocturnal hypoxia, increase in atrial and ventricular transmural pressure, and transient and sustained sympathetic nervous activation (Cowie et al., 2021; Dempsey et al., 2010; Narkiewicz et al., 1999) and is associated with increased risk of systemic hypertension (Logan et al., 2001; Marin et al., 2012), atrial fibrillation and its recurrence (Gami et al., 2004; Neilan et al., 2013; Tung & Anter, 2016), stroke (Valham et al., 2008; Yaggi et al., 2005), and sudden cardiac death during sleep (Gami et al., 2005, 2013). While it is desirable to routinely screen for sleep apnea and to introduce appropriate treatment in patients with cardiovascular diseases, there are practical limitations in terms of cost and effort to implement polysomnography or even type IV portable monitors (Chesson et al., 2003) in a typical clinical setting for cardiovascular disease. Furthermore, it is increasingly recognized that there are limits to the reliability of single‐night sleep study in diagnosis and screening of sleep apnea. A number of studies have reported substantial night‐to‐night variability in apnea–hypopnea index (AHI) in patients with suspected and confirmed sleep apnea (Ahmadi et al., 2009; Gouveris et al., 2010; Prasad et al., 2016; Punjabi et al., 2020; Sforza et al., 2019; Stepnowsky et al., 2004). A meta‐analysis of results from multi‐night sleep studies has revealed a remarkable intra‐individual variability of nightly respiratory parameters leading to a high rate of missed diagnosis of obstructive sleep apnea and varying severity classes from night to night (Roeder et al., 2020). The development of an efficient method for screening sleep apnea across multiple nights that can be used in general clinical practice for cardiovascular diseases is an urgent clinical challenge.

In this study, we applied an ECG‐based screening method of sleep apnea on seven‐day continuous ECG monitoring, which has recently become available in clinical practice. Studies analyzing R‐R interval variability of ECG during polysomnography have reported that the frequency of cyclic variation of heart rate (CVHR) reflects the AHI (Arikawa et al., 2020; Hayano et al., 2011, 2013; Hsu et al., 2020; Magnusdottir & Hilmisson, 2018; Yatsu et al., 2021). CVHR is a characteristic heart rate variability that appears with the episodes of sleep apnea and consists of bradycardia during apnea and transient tachycardia at the cessation of apnea (Guilleminault et al., 1984). As this pattern of heart rate fluctuation repeats itself with the repetitive appearance of sleep apnea episodes, the heart rate also forms a cyclic variation. In a previous study of 864 polysomnographic subjects with suspected sleep apnea, the frequency of CVHR per hour (Fcv) during sleep was correlated with the AHI obtained from the simultaneous polysomnography with *r* = 0.84. When Fcv > 15/h was used as the cutoff, patients with AHI > 15 were detected with a sensitivity of 83% and specificity of 88%. The detection of CVHR in the 24‐h Holter ECG has already been used clinically as a screening method for sleep apnea (Hayano et al., 2017; Shimizu et al., 2015). In this study, we applied this method to seven‐day continuous ECG recordings obtained with an ultrasmall patch ECG recorder with a built‐in three‐axis accelerometer, and detected the CVHR during sleep periods determined from body positions and movements detected by the three‐axis accelerometer. From the obtained CVHR data, we investigated the night‐to‐night dynamic behavior of sleep apnea and its impact on screening for the disease.

## 2 METHODS

### 2.1 Participants

We analyzed data in consecutive 172 outpatients (69 men, 91 women, and 12 of unknown gender; age ± SD, 68 ± 15 years) who underwent seven days of continuous ECG monitoring for the evaluation of suspected cardiac arrhythmias between August 2020 and March 2021 in Japan. The subjects were eligible when they were (1) aged 20 years or older, (2) had provided written informed consent for the use of the anonymized data in this study, (3) were in sinus rhythm in 12‐lead ECG at the entry, and (4) had willingness to comply with up to seven days of continuous ECG monitoring. Subjects were excluded if they had less than four nights of valid sleep ECG data during the monitoring period, where valid sleep ECG data were defined as the total duration of analyzable ECG in sinus rhythm during the time in bed for sleep (TIB), which was at least 80% of the TIB.

The written informed consent was obtained from each subject by JSR Corporation (Japan). The protocol was approved by the Ethics Review Committee of the Nagoya City University Graduate School of Medical Sciences, Nagoya, Japan (No. 60–18–0211).

### 2.2 Device description

ECG and three‐axis acceleration signals were continuously recorded using an ultracompact (30 mm ×100 mm, 5 mm thick, and 12 g in weight), patch‐type, flexible, codeless, electrode‐integrated, and waterproof ECG recorder with a built‐in three‐axis accelerometer (Heartnote®, JSR Corporation, Tokyo, Japan). The recorder was attached on the upper chest wall with adhesive tape. ECG and three‐axis acceleration signals were recorded at 256 Hz and 32 Hz, respectively, for seven days and stored in it.

### 2.3 Data collection

The data collection was carried out in the following steps. The sensors were loaned to clinics, attached to patients, collected after measurements, and returned to JSR Corporation, where stored data were extracted. On a long‐term Holter ECG analysis viewer (NEY‐HEA3000, Nexis Co., Ltd., Fukuoka, Japan), all QRS complexes were detected, and noise and arrhythmia types were annotated using a Holter analyzer program (JMDN 36827012, Nexis Co., Ltd., Fukuoka, Japan; Medical device approval number 228AGBZX00099000, Japanese Ministry of Health, Labour, and Welfare). QRS complexes were classified by the standard cycle length criteria for supraventricular ectopic heartbeats, grouped by morphology, and labeled according to the type of arrhythmia. The results of the automated analysis were reviewed and edited by skilled technicians, and the morphological classification table was provided to medical doctors for confirmation. R‐R interval time series with rhythm annotations were generated for this study.

### 2.4 Dana analysis

#### 2.4.1 Estimation of body posture and body motion intensity

Three‐axis acceleration signals were analyzed to estimate body posture and body motion intensity. First, according to earlier reports (Fortune et al., 2014; Lugade et al., 2014), the acceleration signal was divided into gravitational component and body motion component. Let the calibrated three‐axis acceleration signals be *Ax*(*t*), *Ay*(*t*), and *Az*(*t*). The gravitational components *GCx*(*t*), *GCy*(*t*), and *GCz*(*t*) were obtained by low‐pass filtering the *Ax*(*t*), *Ay*(*t*), and *Az*(*t*) at a corner frequency of 0.25 Hz. The body motion components *BMx*(*t*), *BMy*(*t*), and *BMz*(*t*) were obtained by band‐pass filtering the residuals *Ax*(*t*)‐*GCx*(*t*), *Ay*(*t*)‐*GCy*(*t*), and *Az*(*t*)‐*GCz*(*t*) at 2 to 3 Hz. Second, body postures (supine, right lateral, left lateral, prone, and sitting/standing) were estimated every minute from the direction of body axes determined by *GCx*(*t*), *GCy*(*t*), and *GCz*(*t*), taking into account the direction and angle of the accelerometer with respect to the body axes, which were calculated according to the body positions when the accelerometer was worn in the laboratory. During ambulatory monitoring, lying down was determined when the cranio‐caudal axis was between 50 and 130 degrees with the direction of gravity as 0 degree (Lugade et al., 2014). Upright postures (sitting or standing) were determined when the cranio‐caudal axis was <50 degrees. Among lying postures, supine and prone positions were determined when the antero‐posterior axis was <45 degree and >135 degree, respectively, and when it was between 45 and 135 degrees, the right and left lateral positions were determined when the right‐to‐left axis was >135 degree and <45 degree, respectively. Third, the average intensity of body motions was estimated every minute as

$$\text{Body}\;\text{motion}\;\text{intensity}=\sqrt{BMx{\left(t\right)}^{2}+BMy{\left(t\right)}^{2}+BMz{\left(t\right)}^{2}}.$$

#### 2.4.2 Estimation of sleep periods and extraction of sleep ECG

The ECG data during sleep (sleep ECG) were identified in the following way. First, a one‐week monitoring period was divided into 24‐h intervals from the beginning. Second, based on the analysis of three‐axis acceleration signal, if the subject was judged to have been resting in the lying position (including supine, lateral, prone, and slightly raised upper body positions) with average body motion intensity <10 mG for more than three consecutive hours (brief <3‐min periods of standing/upright position in between were acceptable), that period was defined as TIB. When there were multiple TIBs during a 24‐h segment, only the longest one was defined as the TIB for the segment. When a TIB spanned two 24‐h segments, it was treated as a single TIB belonging to the former 24‐h segment. Fourth, ECG data were extracted for each TIB period and only when the ECG had a total duration in sinus rhythm longer than 80% of the TIB period, the ECG data was defined as a valid sleep ECG data. Finally, only those subjects who had four or more valid sleep ECG data during the monitoring period were included for the final study.

#### 2.4.3 Detection of CVHR

R‐R interval time series obtained from valid sleep ECG data were analyzed by the automated CVHR detection algorism named Auto‐Correlated Wave Detection with Adaptive Threshold (ACAT). The detail of this algorithm has been reported previously (Hayano et al., 2011, 2013, 2021). Briefly, the ACAT algorithm is a time‐domain analysis that detects cyclic, autocorrelated dips in R‐R interval time series, evaluates the features of the dips meet the criteria for CVHR, and calculates the hourly frequency of the dips that fit as CVHR. The ACAT algorithm includes the following steps: First, the R‐R interval time series are smoothed by second‐order polynomial fitting, and all dips with widths between 10 and 120 s and depth‐to‐width ratios of >0.7 ms/s are detected. Also, the upper and lower envelopes of the interval variations are calculated as the 95th and 5th percentile points, respectively, within a moving window with a width of 130 s. Second, the dips that met the following criteria are considered CVHR: (1) a relative dip depth > 40% of the envelope range at the point of dip (adaptive threshold), (2) interdip intervals (cycle length) between 25 and 130 s, (3) a waveform similar to those of the two preceding and two subsequent dips with a mean morphological correlation coefficients > 0.4 (autocorrelated wave), and (4) three cycle lengths (L_1_, L_2_, and L_3_) between four consecutive dips that meet the following equivalence criteria: (3‐2L_1_/s) (3‐2L_2_/s) (3‐2L_3_/s) > 0.8, where s = (L_1_+L_2_+L_3_)/3. Finally, the number of dips comprising the CVHR is counted and the mean hourly frequency of the dips is calculated as Fcv.

Based on earlier studies on the relationship between Fcv and AHI (Hayano et al., 2011, 2013), the severity of sleep apnea was estimated from Fcv as four categories: no (<5 cph), mild (≥5 cph Fcv <15 cph), moderate (≥15 cph Fcv <30 cph), and severe sleep apnea (Fcv ≥ 30 cph).

### 2.5 Endpoints

The main endpoints of interest were the presence of two phenomena in Fcv: the first‐night effect of monitoring and night‐to‐night variability during monitoring. Fcv was calculated for each night with valid sleep ECG data. The first‐night effect was evaluated as the systemic difference in Fcv averaged over all participants between nights. The night‐to‐night variability was evaluated using the coefficient of variance (CV), the average and SD of Fcv during the seven days were calculated within each participant, and the CV was calculated in each participant using the following formula.

$$\text{CV}\left(\%\right)=100\times \frac{\text{SD}\;\text{of}\;\text{Fcv}}{\text{Average}\;\text{Fcv}}$$

The other endpoints of interest were the change in the category of estimated sleep apnea severity between nights, probability of misdiagnosis of moderate‐to‐severe sleep apnea, and potential predictors of night‐to‐night variability of sleep apnea, which include gender, age, average Fcv, night‐to‐night variation in the temporal ratio of body position during sleep, and night order (especially, the first‐night effect on the relative deviation from the seven‐day average). The night‐to‐night variation in the temporal proportions of body positions was measured by CV, which is the SD of the proportion of time spent in a body position divided by the average proportion over seven days.

### 2.6 Statistical analysis

SAS program package version 9.4 (SAS Institute, Cary, NC) was used for statistical analyses. The first‐night effect was examined by a repeated‐measures ANOVA with the mixed procedure adjusted for the effects of age and gender. Linear regression analysis was used to investigate the associations of the average and CV of Fcv variation with age. Nonlinear associations of the CV of Fcv variation with the average Fcv were analyzed with the logarithmic term of average Fcv. Differences in average Fcv with body position during sleep were evaluated by the General Linear Model procedure adjusting for the effects of age and gender. To examine whether night‐to‐night variation in Fcv is due to night‐to‐night variations in temporal ratio of each body position during sleep, the relationships between the CV of Fcv and the CV of temporal ratio of each body position during seven days were analyzed with linear regression. Statistical significance was evaluated at a type 1 error level of 0.05 with the Bonferroni adjustment according to preplanned hypothesis.

## 3 RESULTS

### 3.1 Characteristics of participants

Of the 172 patients who met the inclusion criteria, 52 patients were excluded because they did not have at least four valid sleep ECGs during the monitoring period. The reasons for not obtaining the required number of valid sleep ECG were excessive noises in 40 (23%) patients, long episode(s) of atrial fibrillation in 5 (3%) patients, and the other frequent arrhythmias in 7 (4%) patients. The remaining 120 (70%) patients were included in the final analysis (Table 1).

**TABLE 1:** Patients' characteristics and average sleep parameters during 7‐day continuous monitoring

*N* (%)	120
Age, year	65 ± 15
Gender (men/women/unknown)	44 (37%) / 67 (55%) / 9 (8%)
Length of monitoring, day	7 (7–7)
Number of consecutive days used to detect CVHR, day	6 (5–7)
Number of sleeps analyzed	6 (5–7)
TIB, min	463 ± 76
Time in supine position^a^, %	52 ± 26
Time in right‐recumbent position, %	24 ± 25
Time in left‐recumbent position, %	18 ± 18
Time in prone position, %	6 ± 10

Note

Data are frequency (%), mean ± SD, or median (IQR).

Abbreviations: SPT, sleep period time; TIB, time in bed; TST, total sleep time; WASO, wake time after sleep onset.

Including time in position with the upper body slightly raised.

### 3.2 Fcv during seven‐day monitoring

Table 1 also shows the average number of sleeps during the monitoring periods, TIB, and the percentages of time spent in each body position during sleep estimated from the 3‐axix acceleration data.

**FIGURE 1:**
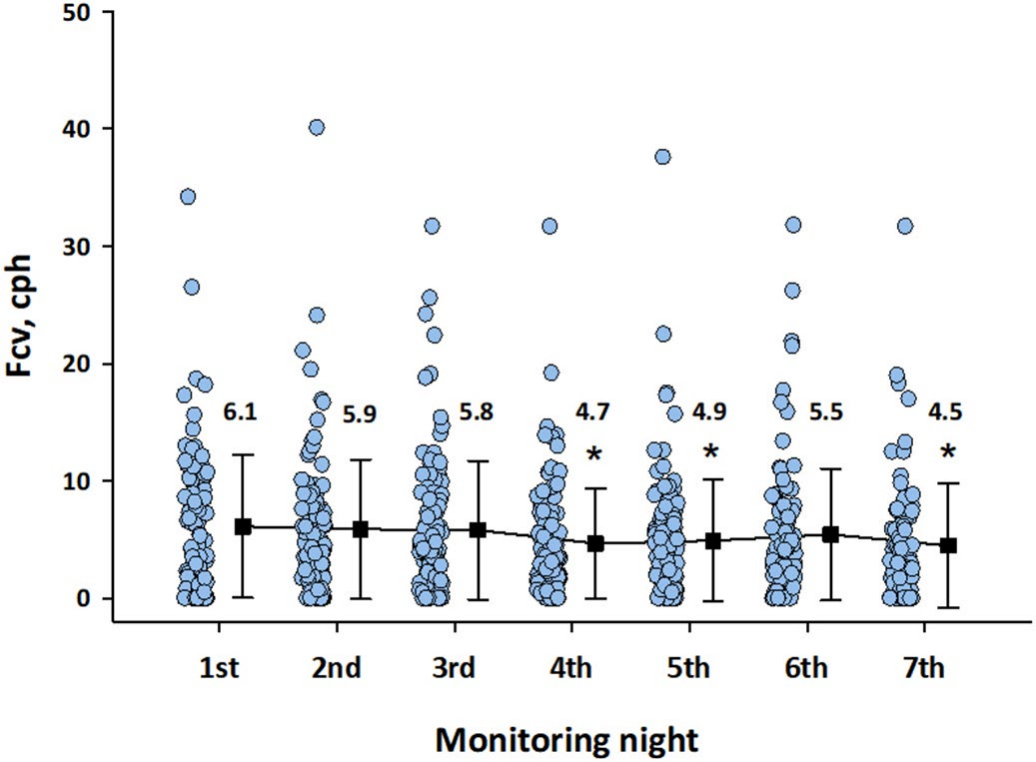
Changes in the frequency of cyclic variation of heart rate (Fcv) across all participants during seven days. Open circles indicate the Fcv in individual participants for each night. Closed squares with error bars indicate the mean and SD of all participants for each night. The value is the mean Fcv (cph) for each day. *Significantly differs from the average of the first night. cph, cycle per hour

Figure 1 shows the changes in Fcv across all participants for each of seven nights. The repeated measures ANOVA revealed that the Fcv differed significantly between seven nights (*p* = 0.0004) and that the Fcv on the first night was slightly but significantly greater than those on nights 4, 5, and 7, indicating a significant first‐night effect of monitoring. Table 2 shows the average and variability of Fcv detected in all valid sleep ECGs during the seven‐day monitoring and in the ECGs excluding that of the first night. Regardless of the effects of the first night, the average Fcv was greater in men than in women.

**TABLE 2:** Frequency of cyclic variation of heart rate (Fcv) during 7‐day monitoring

	Total (*N* = 120)	Subjects with known gender		
		Men (*N* = 44)	Women (*N* = 67)	p*
Including the first night				
Average, cph	5.2 ± 4.7	7.5 ± 5.9	4.5 ± 2.8	0.0002†
CV, %	66 ± 35	58 ± 33	73 ± 37	0.8‡
Range (max‐min), cph	6.5 ± 4.3	7.7 ± 4.8	5.4 ± 3.3	0.2‡
Excluding the first night				
Average, cph	5.1 ± 4.7	6.9 ± 6.1	3.7 ± 3.0	0.0002†
CV, %	66 ± 39	55 ± 36	74 ± 40	0.6‡
Range (max‐min), cph	5.8 ± 4.2	6.7 ± 4.7	4.9 ± 3.1	0.5‡

Note

Values are mean ±SD. *Significance of difference among subjects with known gender. †Significance of difference between men and women adjusted for the effect of age. ‡Significance of difference between men and women adjusted for the effects of age and log(average Fcv).

Abbreviations: cph, cycle per hour; CV, coefficient of variance.

### 3.3 Night‐to‐night variability of Fcv

Fcv showed a considerable night‐to‐night variability during seven‐day monitoring (Table 2). The CV was 66 ± 35% on average with no significant difference between men and women among subjects with known gender. The night‐to‐night variability of Fcv did not change substantially after excluding data from the first night.

When categorizing estimated severity of sleep apnea using the average Fcv during the seven days, five participants (4%) were estimated to have moderate‐to‐severe sleep apnea, whereas 17 (14%) and two (2%) participants were estimated to have moderate‐to‐severe sleep apnea, when using the highest and lowest Fcv throughout seven days, respectively (Table 3).

**TABLE 3:** Relationships between categories of sleep apnea severity estimated by the highest, lowest, and average Fcv throughout 7 days

	*Lowest Fcv*				Total
	No (0–5 cph)	Mild (5–15 cph)	Moderate (15–30 cph)	Severe (>30 cph)	
Average Fcv					
No (0–5 cph)	71 (59%)	0	0	0	71 (59%)
Mild (5–15 cph)	38 (32%)	6 (5%)	0	0	44 (37%)
Moderate (15–30 cph)	0	3 (3%)	1 (1%)	0	4 (3%)
Severe (>30 cph)	0	0	0	1 (1%)	1 (1%)
Highest Fcv					
No (0–5 cph)	40 (33%)	0	0	0	40 (33%)
Mild (5–15 cph)	60 (50%)	3 (3%)	0	0	63 (53%)
Moderate (15–30 cph)	9 (7%)	6 (5%)	0	0	15 (12%)
Severe (>30 cph)	0	0	1 (1%)	1 (1%)	2 (2%)
Total	109 (90%)	9 (8%)	1 (1%)	1 (1%)	120 (100%)

Note

Shaded cells indicate participants who stayed in the same category in the classification of the two criteria.

Abbreviation: cph, cycle per hour.

When the sleep apnea severity was estimated with Fcv of each night, 63% of the participants switched sleep apnea severity categories during the seven days, and 12.5% of participants even switched between the presence and absence of moderate‐to‐severe sleep apnea (Table 3). In addition, only 37% stayed in the same category throughout the seven days, 56% changed by one category, and 8% changed between two categories.

**FIGURE 2:**
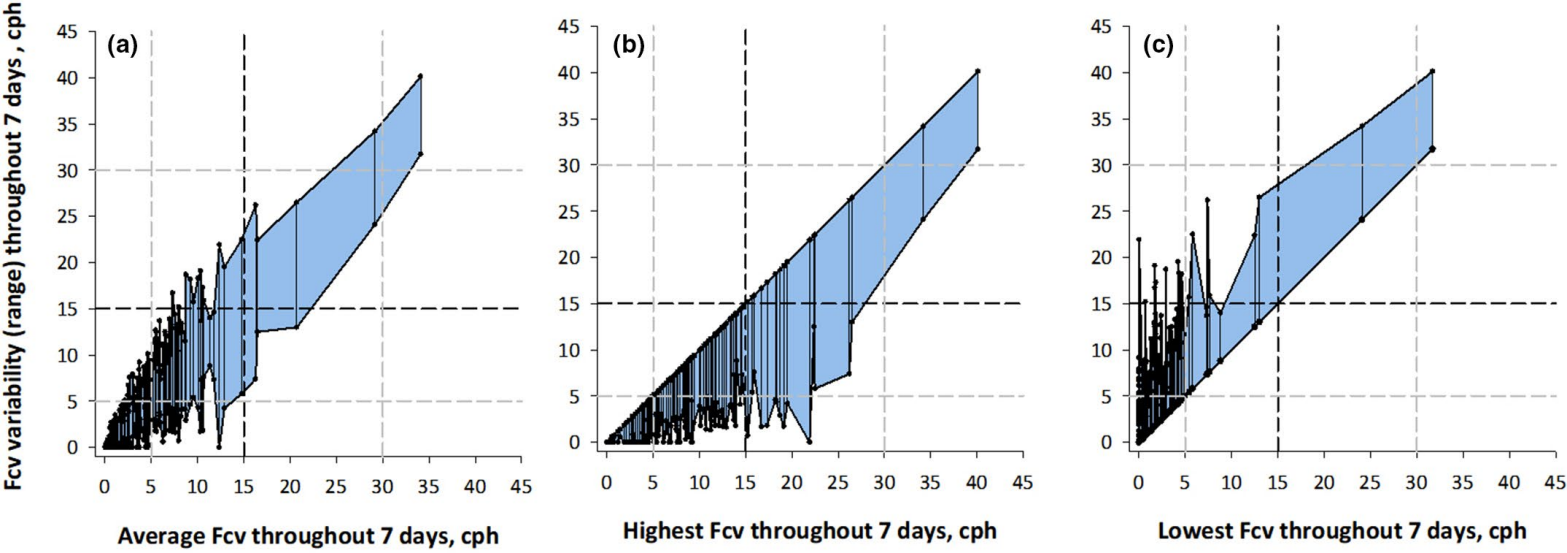
Graphs show the range (maximum and minimum) of night‐to‐night variability in Fcv against the average (panel a), highest (panel B), and lowest (panel c) Fcv throughout seven days. Dashed lines indicate the cutoff values of Fcv for the category of estimated severity of sleep apnea. cph, cycle per hour

Among the 17 patients who were estimated to have moderate‐to‐severe sleep apnea at least one night during the seven days, Fcv was <15 cph on 63 nights (62%) for a total of 101 nights during the seven days, and in 15 out of 17 patients (88%), Fcv was <15 cph on at least one night during the seven days (Figure 2 and Table 3).

### 3.4 Predictors of night‐to‐night variability of Fcv

**FIGURE 3:**
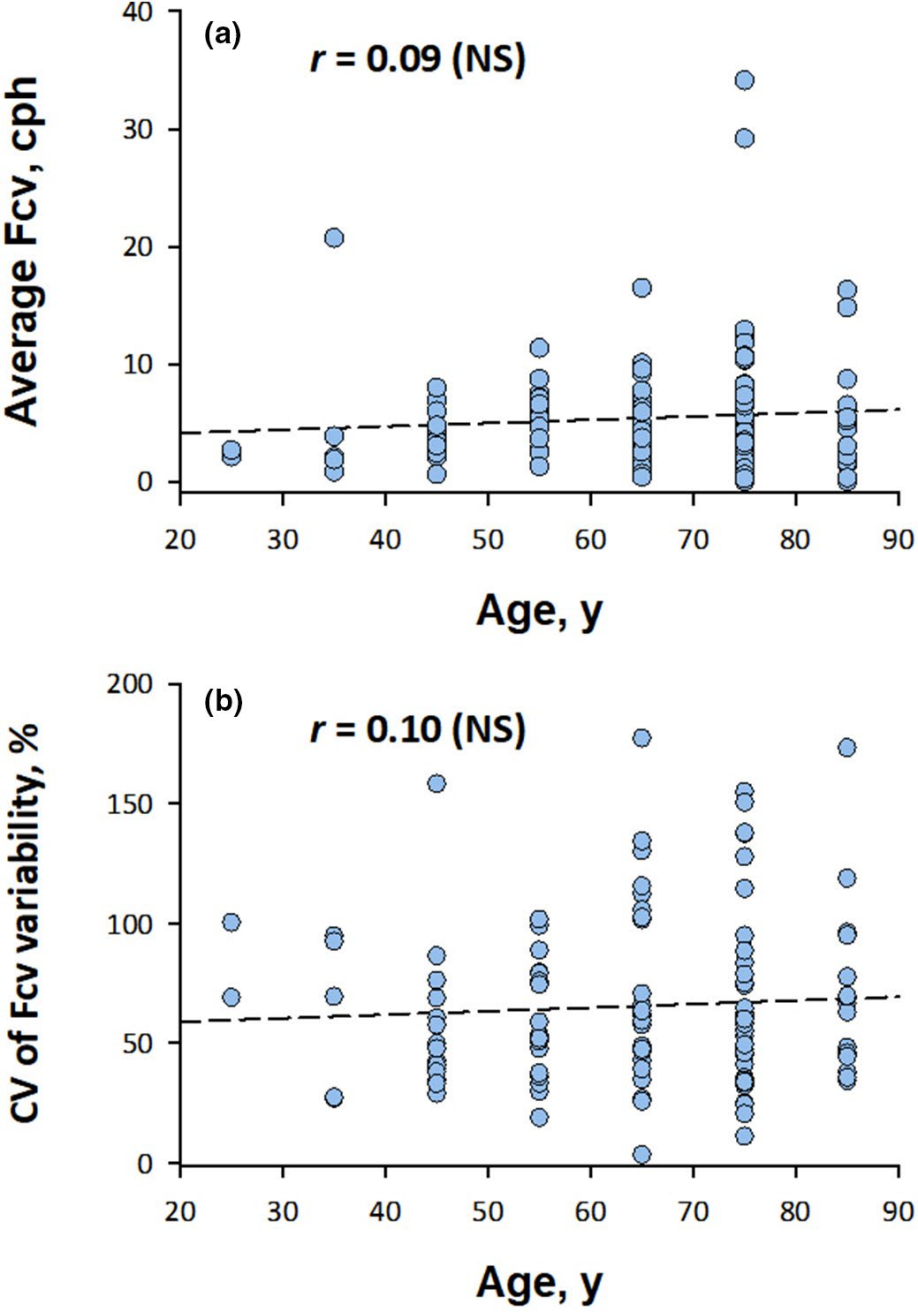
Average and coefficient of variance (CV) of night‐to‐night Fcv variation by age group (*N* = 120). The dashed lines indicate the linear regression lines. cph, cycle per hour

**FIGURE 4:**
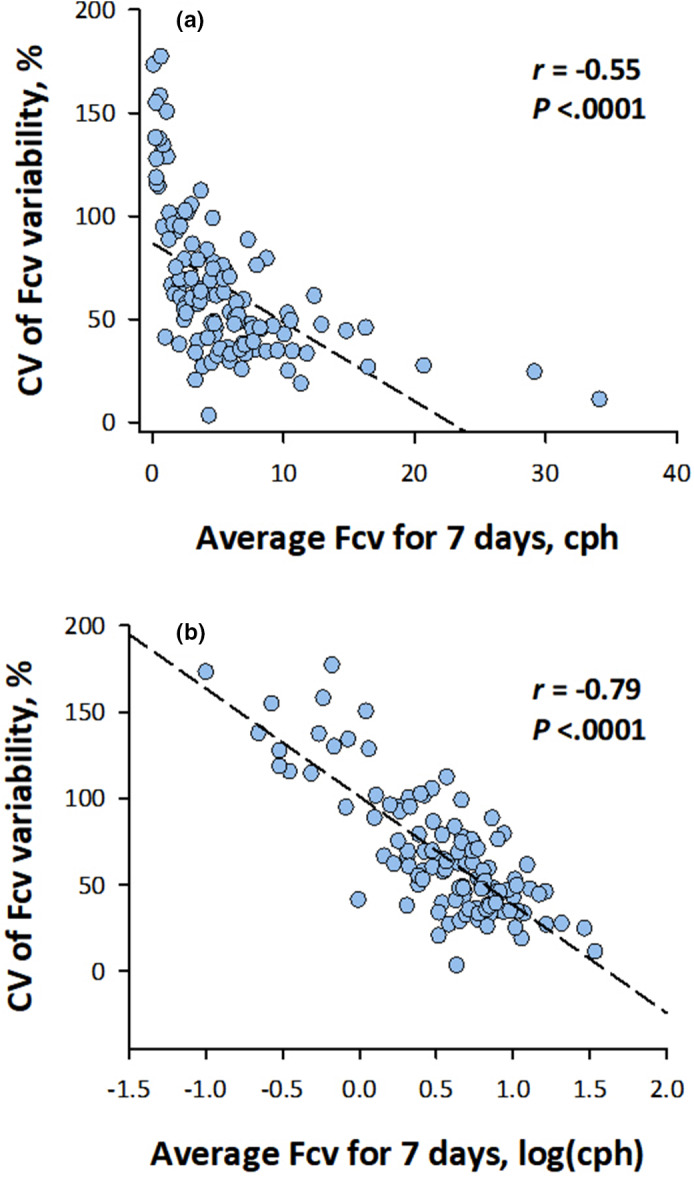
CV of night‐to‐night Fcv variability by average Fcv throughout seven days. *X*‐axis (average Fcv) is linear in panel A and is logarithmic in panel B. The dashed lines represent the linear regression lines. cph, cycle per hour

Neither the average Fcv nor the CV of Fcv variability showed no significant correlation with age (Figure 3). The CV of Fcv during seven days decreased as the average Fcv for seven days increased (Figure 4a). The effect of the average Fcv on the CV of Fcv was better regressed by the logarithmic term of the average Fcv (Figure 4b).

**FIGURE 5:**
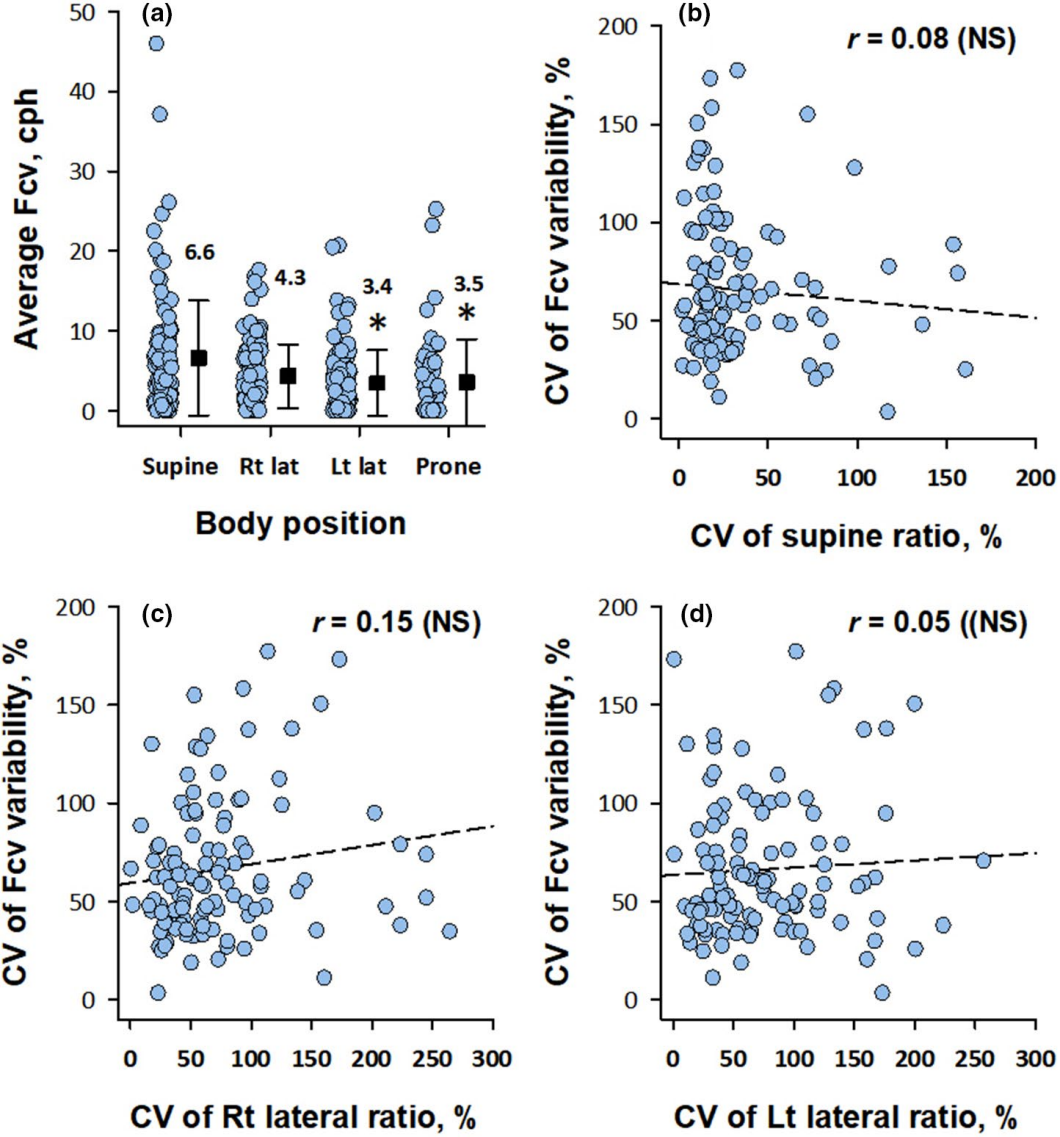
Averages Fcv in each posture during sleep (panel a) and the effect of night‐to‐night variation of percentage of time in each posture on the night‐to‐night variability of Fcv (panels b‐d). In panel a, black closed squares with error bars indicate mean and SD of average Fcv. The value is the mean Fcv (cph) for each position. *Significantly greater than the value in the supine position after the adjustment of the effects of age and gender. In panels B‐D, dashed lines indicate linear regression lines. cph, cycle per hour

When Fcv was compared between body positions during sleep, Fcv was higher in the supine position than in the left lateral and prone positions (Figure 5a) whereas the CV of the night‐to‐night variations in the percentage of time spent in the supine, right lateral, or left lateral postures had no significant effect on the CV of Fcv (Figure 5b‐d), indicating that the night‐to‐night Fcv variability was not due to the night‐to‐night variation in the percentage of body postures during sleep (the percentage of time in the prone position was too low to calculate CV).

**FIGURE 6:**
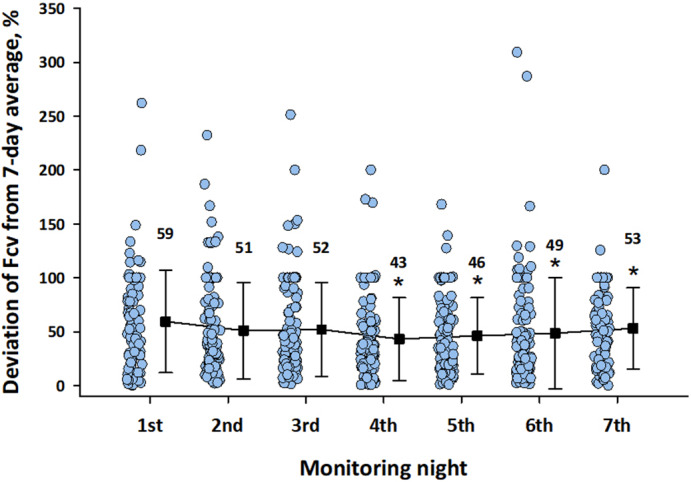
Deviation of Fcv on nights 1 to 7 from average Fcv for seven days. Line graph indicated mean (closed squares) and SD (error bars). The value is the mean value of Fcv deviation (%) for each day. *Significantly smaller than the first‐night value after adjustment for the effects of age, gender, and log (average Fcv)

Finally, we examined whether the variability of Fcv depended on the order of nights. As shown in Figure 6, the deviation of Fcv from the seven‐day average was greatest on the first night and significantly greater than that on fourth, fifth, and sixth nights.

## 4 DISCUSSION

We applied the CVHR‐based screening method of sleep apnea on seven‐day ECG recordings obtained by a patch‐type ECG recorder with built‐in three‐axis accelerometer to investigate the long‐term dynamic behavior of sleep apnea. Fcv was slightly but significantly higher on the first night than on the fourth and subsequent nights, despite ambulatory ECG monitoring at home. Also, Fcv showed considerable within‐individual night‐to‐night variability with an average CV of 66%. When sleep apnea severity was estimated from Fcv of each night, only 37% of participants stayed in the same category of severity through seven days and 67% of participants switched between one or two categories. Among participants estimated to have moderate‐to‐severe sleep apnea (Fcv > 15 cph) at least one night during the seven days, Fcv was <15 cph on 62% of nights, and 88% of the participants had Fcv < 15 cph on at least one night during the seven days, indicating that screening with a single 24‐h Holter monitoring is likely to miss considerable patients with moderate‐to‐severe sleep apnea. In this study, we also examined the possible predictors of night‐to‐night variability of Fcv. While the average Fcv for seven days was higher in men than in women, no significant difference in the night‐to‐night Fcv variability was observed with gender or age. Also, while the average Fcv was higher in the supine position than in the left lateral and prone positions, the night‐to‐night Fcv variability was not dependent on the night‐to‐night variability in body position evaluated with the CV of nightly variation in the temporal proportion of each body position. On the other hand, the relationship between the average and CV of Fcv during seven days (Figure 4) suggested that patients with mild sleep apnea seem to have a higher night‐to‐night variability than in patients with severer sleep apnea. Also, the Fcv variability was higher on the first night than on fourth and subsequent nights. These observations indicate that seven‐day ECG monitoring reveal the night‐to‐night dynamics of sleep apnea and greatly improve the detection of sleep apnea, which may be missed by 24‐h Holter monitoring.

In this study, an algorithm of the auto‐correlated wave detection with adaptive threshold was used to detect CVHR and Fcv was used to estimate AHI. The usefulness of the CVHR as a maker of sleep apnea has been reported by many researchers (Arikawa et al., 2020; Guilleminault et al., 1984; Hayano et al., 2011; Hsu et al., 2020; Magnusdottir & Hilmisson, 2018; Shimizu et al., 2015; Yatsu et al., 2021), and the accuracy of AHI estimation by Fcv has been confirmed by several earlier studies (Hayano et al., 2011, 2013, 2020, 2021). In a study of 864 polysomnographic subjects with suspected sleep apnea, patients with AHI > 15 were detected with a sensitivity of 83% and specificity of 88% by using Fcv > 15 cph as a criterion (Hayano et al., 2011). In another study of 165 male workers, subjects with AHI > 15 were identified with 88% sensitivity and 97% specificity with a criterion of Fcv > 15 cph (Hayano et al., 2013). These studies also reported that the classification performance of Fcv was unaffected by older age (≥ 65 years), obesity, hypertension, diabetes mellitus, dyslipidemia, or the type of sleep apnea (obstructive or central) (Hayano et al., 2011, 2013). The merit of this method is that it requires only a single‐lead ECG (Hayano et al., 2021; Hsu et al., 2020; Magnusdottir & Hilmisson, 2018), and in the present study, by applying this method to long‐term ECG data, we were able to delineate the night‐to‐night dynamics of sleep apnea.

Many studies have reported night‐to‐night variability of sleep apnea in patients with suspected and confirmed sleep apnea (Roeder et al., 2020). The night‐to‐night variability in sleep apnea was observed in both AHI and oxygen desaturation index (ODI) in both laboratory polysomnography and home portable monitoring, and tended to be greater in long‐term monitoring (7–14 days) than in two or three consecutive night studies (Ahmadi et al., 2009; Gouveris et al., 2010; Prasad et al., 2016; Punjabi et al., 2020; Sforza et al., 2019; Stepnowsky et al., 2004; Stoberl et al., 2017). Although the sleep apnea and its severity were estimated by CVHR in the present study, observed night‐to‐night variability in Fcv was comparable to those in AHI and ODI in the earlier studies. In a study of nightly pulse oximetry for two weeks in 77 patients, Stoberl et al. (2017) observed a shift in ODI‐based sleep apnea severity category in 77.9% of patients during the study period. With the meta‐analysis of multi‐night sleep studies, Roeder et al. (2020) reported a linear increase in the proportion of patients with a change in sleep apnea severity class as the number of measurement nights increased. The percentage ranged from 40% at two nights to 70% at seven nights, which is consistent with the 67% with seven‐day measurement in the present study.

In this study, we also analyzed the factors affecting the night‐to‐night variability of Fcv. We found that the seven‐day CV of Fcv was negatively correlated with the seven‐day average of Fcv. Similar observations were reported for ODI during two weeks (Stoberl et al., 2017) and AHI during two to eight consecutive nights (Prasad et al., 2016; Punjabi et al., 2020; Sforza et al., 2019). These indicates that patients with mild sleep apnea show greater night‐to‐night variability than those with severer disease and would benefit from multi‐night sleep studies. Because sleep apnea syndrome is more likely to occur in the supine position than in the lateral or prone positions (Yalciner et al., 2017), the night‐to‐night variability in time spent in the supine position could be a factor of night‐to‐night variability of sleep apnea. This hypothesis has been supported by some studies (Yalciner et al., 2017) but not by others (Chediak et al., 1996). In the present study, we also observed a higher average Fcv in the supine position than in the left lateral and prone positions (Figure 5a), but no significant association was observed between the CV of time spent in a body position and the CV of Fcv (Figure 5b‐d). Finally, the sleep parameters obtained by laboratory polysomnography show the first‐night effects. A meta‐analysis showed that on average, AHI was lower in the first night than in the second night (Roeder et al., 2020). Studies with home portable monitoring reported no consistent result of the systematic changes in AHI or ODI between monitoring nights (Ahmadi et al., 2009; Sforza et al., 2019). In the present study, we observed the higher average Fcv on the first night than on the fourth and seventh nights, but the difference was slight. On the other hand, the deviation of Fcv from the seven‐day average was greater on the first night than on the fourth and subsequent nights, suggesting that the start of monitoring even at home may have affected on the stability of sleep.

The present study has several clinical implications. First, sleep apnea is highly prevalent in patients with cardiovascular diseases and is a factor that worsens the prognosis. Sleep apnea is associated with increased risk of systemic hypertension (Logan et al., 2001; Marin et al., 2012), atrial fibrillation and its recurrence (Gami et al., 2004; Neilan et al., 2013; Tung & Anter, 2016), stroke (Valham et al., 2008; Yaggi et al., 2005), and sudden cardiac death during sleep (Gami et al., 2005, 2013). It is desirable to screen these patients for sleep apnea on a regular basis, but in general clinical practice for cardiovascular disease, there are practical limitations in terms of cost and labor to perform sleep studies, even with home portable monitors. The present study shows that ambulatory ECG recorders, which are well accepted in general practice for cardiovascular diseases, can be used for this purpose. Second, the results of the earlier studies and the present study indicate that sleep apnea shows considerable night‐to‐night variability, and that a single‐night and even two/three‐night sleep testing can lead to a high rate of misdiagnosis and misclassification of disease severity. To achieve good reliability in screening for sleep apnea, multiple sleep studies over a long period of time are desirable, but this is a trade‐off for the burden on patients and clinical resources. Our present results show that seven‐day continuous ECG monitoring using a patch‐type recorder was able to detect night‐to‐night dynamic behavior of sleep apnea, suggesting its potential as a primary screening method for sleep apnea not only in cardiology but also in sleep clinics. Third, in order to detect sleep apnea by continuous monitoring in daily life, it was necessary to identify the temporary positions of sleep periods from data recorded over a long period of time. In addition, information of body posture was useful to assess its impact on sleep apnea. For these purposes, a built‐in three‐axis accelerometer and its fixation to body axis are considered essential for long‐term ECG recorders for this purpose. Finally, analysis of sleep apnea by long‐term continuous electrocardiography is clearly beneficial to patients in that arrhythmia and transient myocardial ischemia can be assessed simultaneously with sleep apnea. In particular, the association between paroxysmal atrial fibrillation and sleep apnea is important in determining strategies for treating both diseases and preventing stroke (Tung & Anter, 2016; Yaggi et al., 2005).

This study has limitations. First, we used Fcv as an estimate of AHI, but its validity was only indirectly ensured by previous studies (Hayano et al., 2011, 2013). Future studies with simultaneous monitoring using established portable sleep apnea monitors are desirable to confirm their validity in the assessment of nocturnal variations in sleep apnea. Second, the study population consisted of consecutive outpatients who underwent seven days of continuous ECG monitoring for screening or evaluation of arrhythmias, but no other information, including underlying medical conditions, such as body weight, alcohol intake, smoking, medications, dental, or otolaryngological parameters, was available. The extensibility of our findings to a variety of clinical conditions needs to be explored in future studies. Third, only a small percentage of the participants in this study had severe sleep apnea. Although the proportion of these patients may be representative to the proportion of the general population undergoing Holter ECG testing, future studies are needed to confirm whether the present results are applicable to patients with severe sleep apnea.

## 5 CONCLUSION

A CVHR‐based screening method of sleep apnea was applied on seven‐day continuous ECG recordings to investigate the long‐term dynamic behavior of sleep apnea. The frequency of sleep apnea estimated from Fcv showed considerable night‐to‐night variability, which was consistent with the night‐to‐night variability of AHI and ODI in earlier studies. It is suggested that the use of seven‐day ECG monitoring can greatly improve the detection of sleep apnea, which may be missed by 24‐h Holter monitoring. CVHR analysis of long‐term ambulatory ECG recordings is useful for improving the reliability of screening for sleep apnea without placing an extra burden on patients with cardiovascular disease and their care.

## CONFLICT OF INTEREST

Heart Beat Science Lab, Co. Ltd. has an advisory agreement with JSR Corporation.

## AUTHOR CONTRIBUTIONS

E.Y. and J.H. conceptualized the study; J.H. contributed to methodology and software; E.Y. and J.H. validated the study; E.Y. involved formal analysis; E.Y. investigated the study; J.H. and E.Y. contributed to resources; J.H. curated the data; J.H. wrote—review and editing; J.H. visualized the study; J.H. contributed to project administration; J.H. and Y.E. involved in funding acquisition. All authors have read and agreed to the published version of the manuscript.

## ETHICAL APPROVAL

The protocol of study was approved by the Ethics Review Committee of the Nagoya City University Graduate School of Medical Sciences, Nagoya, Japan (No. 60–18–0211). Based on the protocol, informed consent for the research use of the anonymized ECG data was obtained from each subject by JSR Corporation (Japan).

## Data Availability

The data that support the findings of this study are available on request from the corresponding author. The data are not publicly available due to privacy or ethical restrictions.
